# The Hadal Amphipod *Hirondellea gigas* Possessing a Unique Cellulase for Digesting Wooden Debris Buried in the Deepest Seafloor

**DOI:** 10.1371/journal.pone.0042727

**Published:** 2012-08-15

**Authors:** Hideki Kobayashi, Yuji Hatada, Taishi Tsubouchi, Takahiko Nagahama, Hideto Takami

**Affiliations:** 1 Institute of Biogeosciences, Japan Agency for Marine-Earth Science and Technology (JAMSTEC), Yokosuka, Japan; 2 Department of Food and Nutrition, Higashi-Chikushi Junior College, Kitakyusyu, Fukuoka, Japan; University of Aberdeen, United Kingdom

## Abstract

The Challenger Deep in the Mariana Trench is the deepest point in the ocean (10,994 m). Certain deep-sea animals can withstand the extreme pressure at this great depth. The amphipod *Hirondellea gigas* is a resident of the Challenger Deep. Amphipods are common inhabitants at great depths and serve as scavengers. However, there is relatively little information available regarding the physiology of *H. gigas* or this organism's ecological interactions in the hadopelagic zone. To understand the feeding behavior of this scavenger in the deepest oligotrophic hadal zone, we analyzed the digestive enzymes in whole-body extracts. We describe the detection of amylase, cellulase, mannanase, xylanase, and α-glycosidase activities that are capable of digesting plant-derived polysaccharides. Our identification of glucose, maltose, and cellobiose in the *H*. *gigas* extracts indicated that these enzymes function under great pressure *in situ*. In fact, the glucose content of *H. gigas* averaged 0.4% (w/dry-w). The purified *H*. *gigas* cellulase (HGcel) converted cellulose to glucose and cellobiose at an exceptional molar ratio of 2∶1 and efficiently produced glucose from dried wood, a natural cellulosic biomass, at 35°C. The enzyme activity increased under a high hydrostatic pressure of 100 MPa at 2°C, conditions equivalent to those found in the Challenger Deep. An analysis of the amino acid sequence of HGcel supported its classification as a family 31 glycosyl hydrolase. However, none of the enzymes of this family had previously been shown to possess cellulase activity. These results strongly suggested that *H. gigas* adapted to its extreme oligotrophic hadal oceanic environment by evolving digestive enzymes capable of digesting sunken wooden debris.

## Introduction

In 1960, the bathyscaphe Trieste voyaged to the bottom of the Challenger Deep in the Mariana Trench, the deepest point in the ocean (10,994 m), where its crew observed certain organisms that could withstand extreme pressure [1]. Some deep-sea animals were caught from deep-sea trenches including the Mariana Trench [2]. The amphipod *Hirondellea gigas* is a resident of this deepest hadal zone [3]–[6]; the species name “*gigas*” refers to the organism's distinctively large body size among the amphipods. Amphipods are common inhabitants of great depths and serve as scavengers [7]–[10], even though the amount of organic carbon or biomass decreases with depth [11], [12]. Not surprisingly, the average body size in the benthos also decreases with increasing depth and/or decreasing food availability [13]–[15]. Thus, it remains unclear how *H. gigas* can survive under extremely high pressures and grow so large despite the oligotrophic environment. Studies of *H*. *gigas* were facilitated when, in 1998, the remotely operated submersible ‘Kaiko’ captured over 100 individuals [3], [4] (Movie S1). Although more than half a century has passed since the discovery of *H*. *gigas*, there is still relatively little information about this organism's physiology and ecological interactions in the hadopelagic zone [16]–[20]. We report the use of cellulase and hemi-cellulase as digestive enzymes by *H. gigas* and the presence of large amounts of the products of these enzymes, glucose and disaccharides, in the bodies of these amphipods. Of particular interest is the finding that the cellulase of *H. gigas* exhibited a novel reaction system that could produce glucose directly from sawdust, crystal cellulose and carboxymethyl cellulose. In fact, some plant and wood debris was found in deep-sea, and hadal trench [21]–[24]. Lemche et al. reported existence of large pieces of wood, coconuts shell as well as blades of sea grass in Palau Trench at the depth of 8021–8042 m [25]. Our results indicate that *H. gigas* could use plant debris as a carbon and energy source to survive in the deepest hadal zone in the world.

## Results

**Figure 1:**
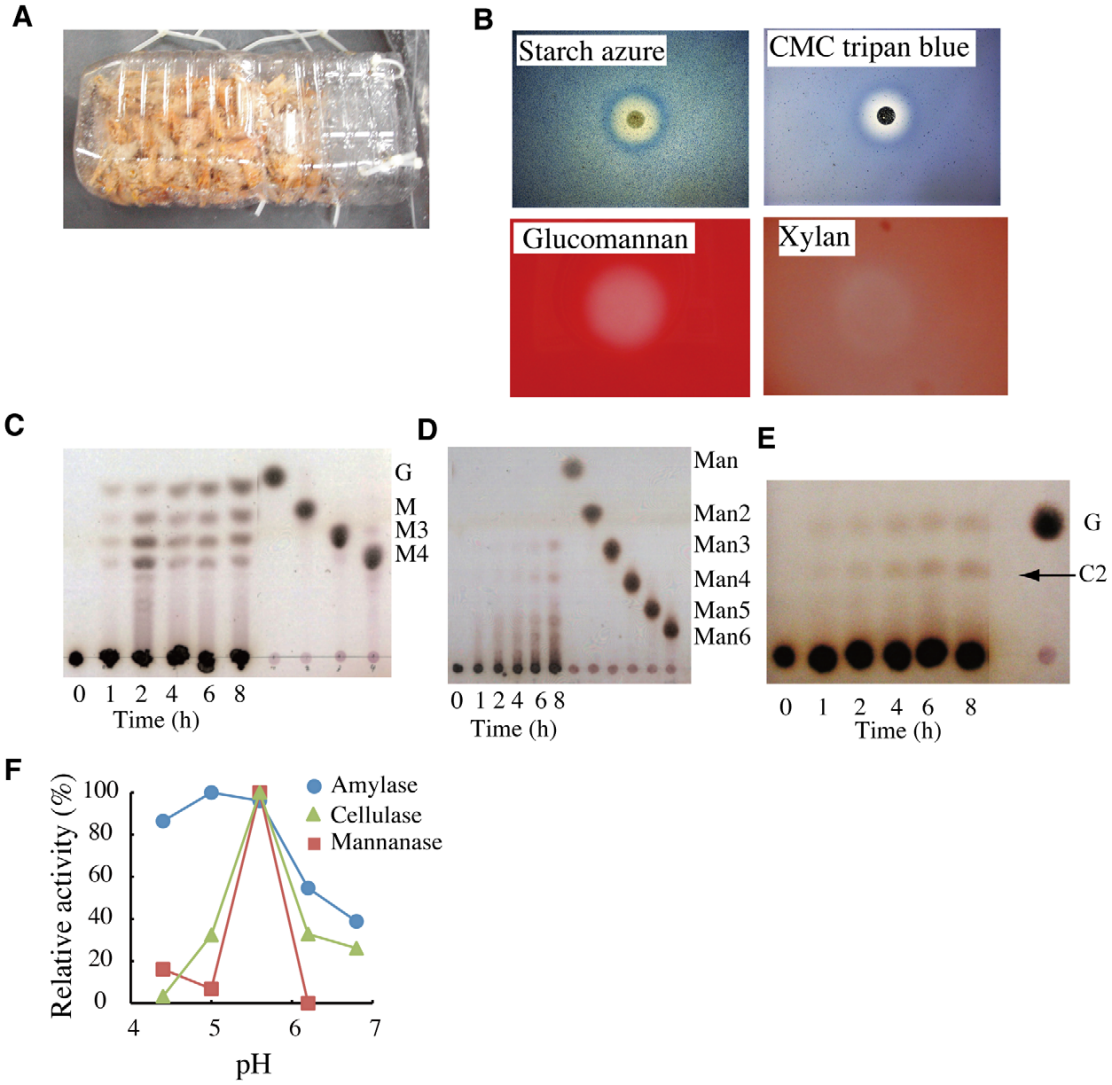
*H*. *gigas* possesses polysaccharide hydrolase activities. We captured deep-sea animals using baited traps containing a slice of mackerel. One baited trap contained approximately 50 individuals (A). Digestive enzyme activities were assessed by halo formation in agar plates containing starch azure (amylase), CMC and trypan blue (cellulase), glucomannan (mannanase), and xylan (xylanase). The halos produced by the amylase and cellulase activities were visualized directly, while the halos resulting from mannanase and xylanase were detected after staining with 0.5% Congo red followed by washing with DDW (B). The kinetics of the reactions were determined by TLC as described in the Methods section of this paper. Reactions with crushed *H*. *gigas* with 0.5% (w/v) starch (C), 0.2% (w/v) glucomannan (E) or 1% (w/v) CMC (D) were conducted in 100 mM sodium acetate buffer (pH 5.6) at 30°C. The pH dependencies of the amylase, mannanase, and cellulase activities were measured with protein extracts (F). The enzyme reactions were conducted in 100 mM sodium acetate buffer (pH 4.4–5.6) or 100 mM sodium phosphate buffer (pH 6.2–6.8). The relative activities are shown.

To understand how *H*. *gigas* thrives at the greatest ocean depths, we lowered an 11,000-m class free-fall sediment sampler with a camera system, ‘ASHURA’ (Fig. S1), into the Challenger Deep on July 10, 2009, to observe this amphipod and to capture specimens in baited traps (11°22.11′N, 142°25.86′E, depth of 10,897 m). We captured 185 individuals during 3 h. The amphipods showed the same morphology as the *H. gigas* specimens captured in 1998 [3]. *H*. *gigas* was the only animal captured (Fig. 1A), and the individuals captured ranged from 2–5 cm in length and 0.3–0.6 g in dry weight. We assayed crushed individuals for the activity of digestive enzymes by observing halo formation on agar plates containing various substrates. We observed halos on the plates that contained starch, carboxymethyl cellulose (CMC), glucomannan and xylan (Fig. 1B). Furthermore, amylase, cellulase, mannanase, xylanase, α-glucosidase and protease activities were measured in five randomly selected individuals (Table 1). We found 1.5- to 5-fold differences in the activity levels between individuals. Although such polysaccharide hydrolases have been shown to digest cellulose and hemicellulose derived from trees [26], we did not detect lignase activity (data not shown). We also detected no such enzyme activity in the bait used in the traps.

**Table 1:** The digestive enzymatic activities detected in *H. gigas* whole-body extracts.

Sample ID	Amylase (mU)	Cellulase (mU)	Mannanase (μU)	Xylanase (μU)	α-Glycosidase (μU)	Protease (mU)
1	112.2	3.42	8.22	0.41	22.2	0.26
2	94.3	2.40	16.1	0.37	17.1	0.27
3	65.5	2.26	39.8	0.36	15.3	0.17
4	65.4	2.25	30.2	0.43	13.7	0.17
5	93.8	3.22	16.6	0.40	27.6	0.23

(per 1 µg of protein)

We characterized the enzymatic products of amylase, mannanase, cellulase, and xylanase by thin layer chromatography (TLC) using an 80% saturated ammonium sulfate precipitate of crushed *H*. *gigas*. The *H. gigas* amylase produced glucose, maltose, maltotriose, and maltotetraose from potato starch at 30°C (Fig. 1C). The mannanase digested glucomannan to produce many low molecular weight polysaccharides. The glucomannan digestion pattern was characteristic of a typical *endo*-polysaccharide hydrolase (Fig. 1D), but neither galactomannan nor curdlan was detectably digested (data not shown). The cellulase produced glucose and cellobiose from CMC (Fig. 1E). Conversely, the xylanase activity was too weak for detection by TLC, and the enzymatic activity was unstable in the crushed individuals. No agarolytic activity on β-D-galactose and 3, 6-anhydro-α-L-galactose polysaccharide derived from seaweed was detected [26]. We initially expected that the production of glucose from CMC was due to cellobiohydrolase and β-glucosidase, as in termites, wood-eating cockroaches and other invertebrates [27]–[30]. However, no β-glucosidase activity (glucose from cellobiose) was detected. Therefore, the polysaccharide hydrolases within *H*. *gigas* appear to produce glucose directly from CMC, a phenomenon not previously observed. Moreover, known polysaccharide hydrolases are typically most catalytically active at a pH between 5.2 and 6.0 and lose their activity when the pH is increased to 8.0 (Fig. 1F). However, the pH of the Challenger Deep is 8.0, suggesting that the polysaccharide hydrolases of *H*. *gigas* are active at much higher pH values than expected. The ability of these enzymes to hydrolyze the cellulose and hemicellulose from trees strongly implies that *H*. *gigas* derives nutrients from tree remnants found in the oceanic depths.

**Figure 2:**
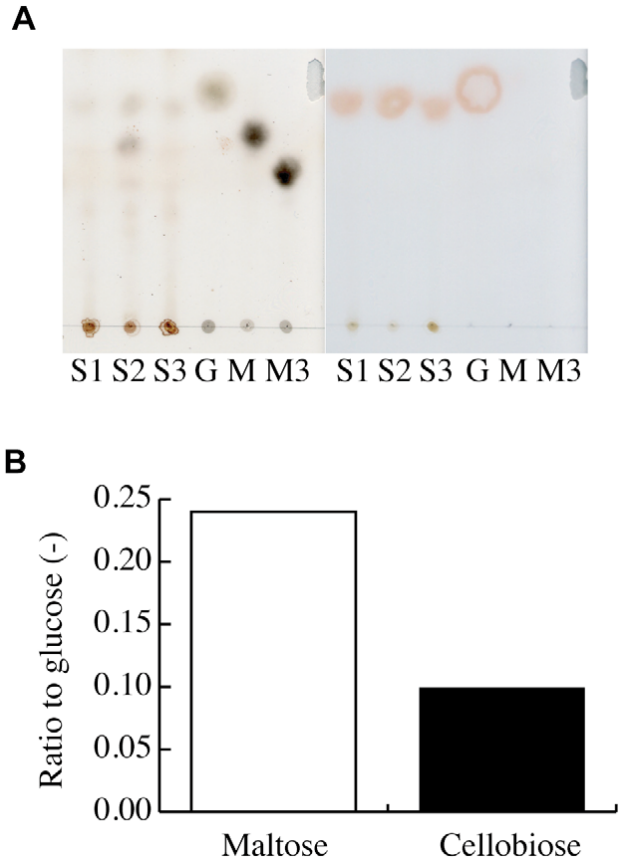
Identification of oligosaccharides in *H*. *gigas* whole-body extracts. Oligosaccharides were extracted with DDW from 3 crushed *H*. *gigas* and were separated by TLC and stained with H_2_SO_4_ for oligosaccharides (A, left) or the Glucose CII Kit for glucose (A, right). Glucose (G), maltose (M), and maltotriose (M3) were used as standard. The maltose and cellobiose contents were measured by the increase in the glucose content after α- or β-glucosidase treatment (B).

To confirm this hypothesis, we observed the gut contents of *H. gigas* under a microscope. In addition to some small pieces of mackerel tissue used as bait in the trap, we found a stick-like material; however, it was difficult to identify this stick-like material as plant debris (Fig. S2). Furthermore, it was difficult to determine from microscopic observation whether the plant debris was digested in the gut. Thus, we determined the oligosaccharide composition of *H*. *gigas* to detect polysaccharide hydrolase digestive products. The two individuals examined contained glucose and disaccharides, which corresponded to the observed polysaccharide hydrolase activities (Fig. 2A). The average glucose content was 0.43±0.1% (w/w) (dry weight) (n = 5), but the disaccharide content varied. We, therefore, determined the disaccharide composition of 30 individuals and found that maltose and cellobiose represented 35% and 17% of the glucose content, respectively (Fig. 2B). The detection of cellobiose strongly supports our hypothesis that *H*. *gigas* naturally digests cellulose. Furthermore, we found several pieces of plant debris in the sediment of the core sampler attached to ASHURA (Fig. S3). In this study, the total organic carbon (TOC) content in the sediment samples (0–15 cm below the surface) was very low (3.22 ppm/g of dry sediment at 11°22.140′N, 142°25.756′E and a depth of 10,895 m; 5.68 ppm/g of dry sediment at 11°22.3130′N, 142°25.9412′E and a depth of 10,867 m). Because the average glucose content of *H*. *gigas* was 1.57 mg (n = 5), the TOC contained in the sediments would be too low to explain the organic carbon content of *H*. *gigas*, indicating that the organism could not derive its carbon from the sediment alone. Although we cannot identify the wooden debris, we did observe several *H*. *gigas* individuals with sediment-filled guts (data not shown). We suggest that *H*. *gigas* is an omnivore that can eat marine snow and other detritus, animal corpses, and driftwood or plant debris.

**Figure 3:**
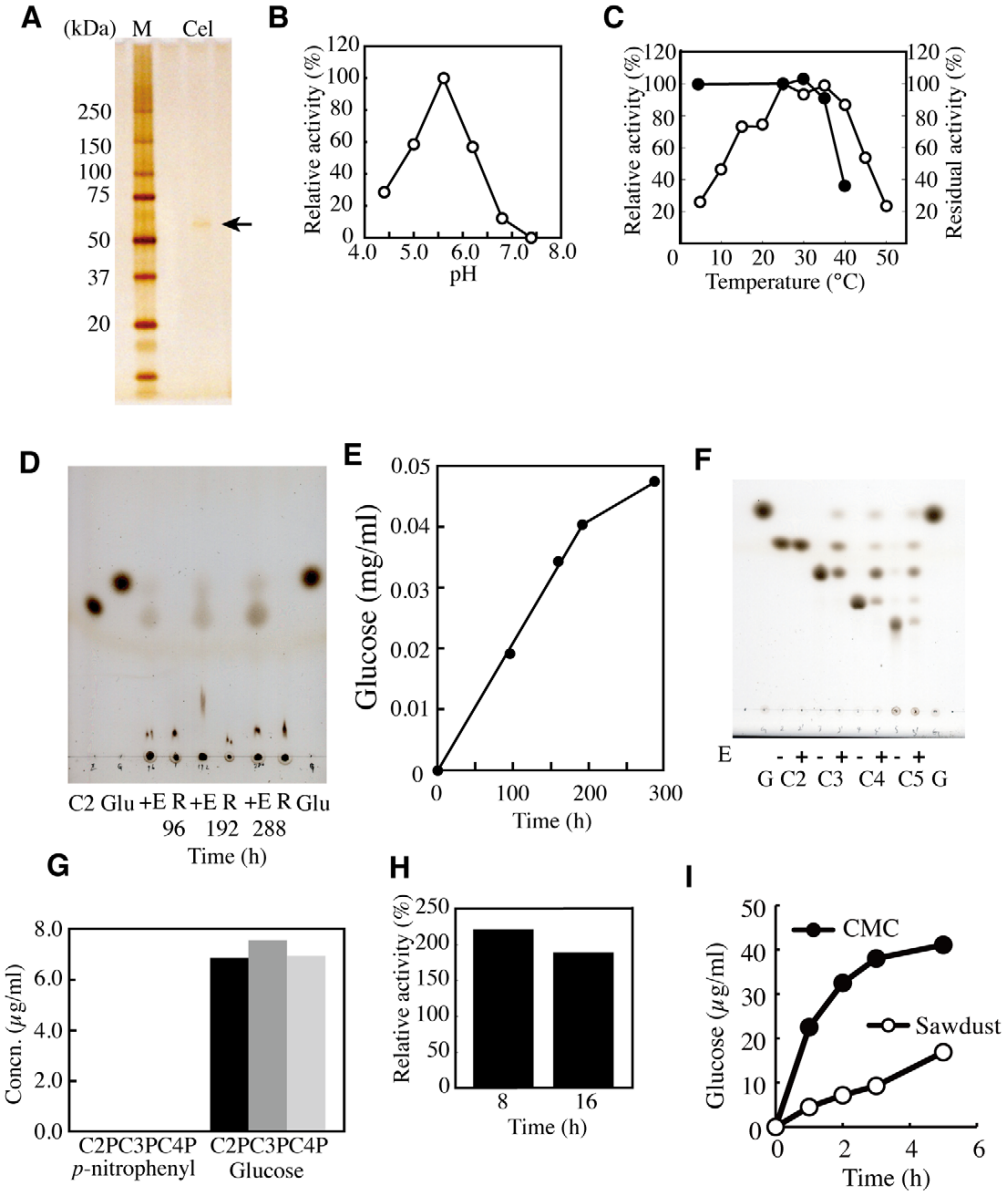
Purification of HGcel. HGcel was purified from crushed *H*. *gigas* by anion-exchange column chromatography as described in the Methods section. HGcel migrated as a single 59-kDa band on an SDS-PAGE gel (5–20% gradient) (A). The effects of pH (B) and temperature on the enzyme's activity and stability are expressed relative to their maximum respective values (C). HGcel converted cellulose to glucose (Glu) and cellobiose (C2). In these reactions, 200 mU of HGcel was added to 500 µl of 5% cellulose solutions (pH 5.6) (D). One of the 5% (w/v) cellulose suspensions was added to HGcel (+E). Another was not added and was used as a reference (R). The kinetics of the glucose production from cellulose are described in the Methods section of this paper (E). The products of the HGcel reaction with cellobiose (C2), cellotriose (C3), cellotetraose (C4), and cellopentaose (C5) were analyzed using TLC. Addition of HGcel (12 mU) is indicated by a ‘+’. HGcel was more efficient at hydrolyzing cello-oligosaccharides larger than cellotriose (F). HGcel (12 mU) was allowed to react with *p*-nitro phenyl cello-oligosaccharides at 35°C for 1 h. *p*-nitro phenyl binds to the reducing ends of cello-oligosaccharides. The release of glucose or *p*-nitro phenol in the reactions is shown (G). The effect of hydrostatic pressure (100 MPa) on the enzymatic activity is expressed as the percentage of its activity at atmospheric pressure (0.1 MPa) (H). The enzymatic reaction was performed using 10 mU of HGcel with 1% CMC solution in airtight plastic tubes at 2°C. The enzymatic activities were measured after 8 h and 16 h of incubation. The kinetics of sawdust digestion by HGcel were measured by determining the production of glucose in a reaction containing HGcel (380 mU) and either sawdust or CMC at 35°C (I).

We next focused on the novel HGcel because of its similarity to the main digestive enzyme of herbivorous animals [28]–[30]. We purified HGcel from 10 individuals with standard salt precipitation and anion exchange techniques. The molecular mass of the purified HGcel was 59 kDa by SDS-PAGE (Fig. 3A, Fig. S4). The optimum pH for cellulose hydrolysis was found to be 5.6 (Fig. 3B), and no enzymatic activity was detected at pH 7.8 (the same pH as on the ocean floor). The optimum activity occurred at 25–35°C at a pH of 5.6 (Fig. 3C). The enzyme retained more than 20% of its activity at 4°C, was stable at 35°C but started to lose activity at 40°C (Fig. 3C). Because the N-terminus of the enzyme was blocked, we determined the internal amino acid sequence with mass spectroscopy of the tryptic peptides. The amino acid sequences of the three peptides, designated P1, P2 and P3, were identified as TPPMGWLAWER, SQMALWAIMAAPLFMSNDL and AVIAVNQDPLGIQGR, respectively. The P1 and P2 sequences were identical to those of diverse alpha-N-acetylgalactosaminidases [*Macaca mulatta* (Accession No. XP_001117342) and an alpha-N-acetylgalactosaminidase-like protein of *Oreochromis niloticus* (Accession No. XP_001117342)] and the glycosyl hydrolase family (GHF) 31 of *Drosophila virilis* (Accession No. XP_002059881.1), respectively [31]. Aligning the P3 sequence with the sequences of alpha-N-acetylgalactosaminidases indicated the presence of one or two deletions or mismatches in the P3 sequence [32]; the known cellulases belong to fourteen different families (GHF 5, 6, 7, 8, 9, 10, 12, 26, 44, 45, 48, 51, 61 and 74) [33], [34]. HGcel produced glucose and cellobiose from CMC at a molar ratio of 2∶1, digested crystal cellulose to produce glucose and cellobiose (Figs. 3D, E), and degraded cello-oligomers larger than cellotriose to produce glucose (Fig. 3F). In addition, HGcel produced glucose from cellobiose, cellotriose and cellotetraose and coupled the reducing ends of these products to *p*-nitro phenyl; we did not detect the absorbance of *p*-nitro phenol (Fig. 3G). We conclude that HGcel represents a novel *exo*-cellulase that degrades the non-reducing end of cellulose to produce glucose and cellobiose. This hydrolysis pattern differs from the *endo*-β-glucanases (EC 3.2.1.4), cellobiohydrolases (EC 3.2.1.91) and β-glucosidases (EC 3.2.1.21) of known herbivorous animals and microorganisms [32], [33]. We attempted PCR amplification of the partial cellulase gene based on the peptide sequences. However, we did not detect distinct DNA bands due to the digestion of the genomic DNA of *H. gigas* during the process of retrieving the samples from the sea bottom.

## Discussion

A single enzymatic pathway that efficiently converts cellulose to its component nutrients would be of high survival value to an organism residing at great oceanic depths. We were able to demonstrate that the enzymatic activity of HGcel increased under high hydrostatic pressure (100 MPa at 2°C). These conditions approximate the natural habitat of *H. gigas* (Fig. 3H). The retrieval of a piece of wood from the ocean floor prompted us to determine whether HGcel could digest wood. The data demonstrate that HGcel degraded wood to glucose at 35°C (Fig. 3I). HGcel also produced glucose and cellobiose from sawdust and CMC at the same 2∶1 molar ratio; the glucose produced from wood was approximately one-fifth of the amount generated from CMC. In addition, HGcel can produce glucose from plain paper at room temperature (Fig. S5). This enzymatic property of HGcel would help *H. gigas* to utilize various cellulosic materials in the oligotrophic environment as carbon sources. Furthermore, HGcel may be useful in the production of glucose from various cellulosic biomass sources to be used in bio-ethanol fermentation [35].

The origin of HGcel poses a significant unanswered question. There are many examples of animals that have acquired cellulases and hemicellulose hydrolases from symbiotic bacteria, fungi or protozoa [36]–[38] during evolution. The bacteria isolated from *H*. *gigas* or Challenger Deep sediments barely grow under 100 MPa at 2°C [39]–[41]. We attempted to detect bacterial, archaeal or eukaryotic rDNA during both our previous study in 1996 and our present study by employing PCR to amplify rDNA from the *H. gigas* samples; we also attempted to isolate microorganisms from *H*. *gigas* itself. However, we were unable to detect any meaningful bacterial or archaeal rDNA PCR signals or to isolate any microbes from *H*. *gigas* (Fig. S6), leading us to conclude that HGcel is a product of intrinsic evolution. We note that the majority of *H*. *gigas* specimens isolated from the Philippine Trench (depth: 9,600–9,800 m) contained bacteria-like cells in their guts [8]; however, these cells could not be definitively identified as symbiotic organisms or bait contaminants. The high hydrostatic pressure of the hadal zone acts as a barrier to other organisms, and the relative isolation of *H*. *gigas* can be assumed to have played a role in the evolution of HGcel.

The deep-sea gastropods utilized the fallen plants as well as wood as the nest [42], [43]. Our results showed the hadal amphipod utilized the fallen plants, crushed small enough to enter its mouth as food to survive in the oligotrophic environment of the deepest trench. Amphipods are one of the most trophically diverse taxa in the marine environment [44]. Blankenship LE and Levin LA investigated the trophic level of amphipods captured from the hadal zones of the Tonga Trench and Kermadec Trench using stable isotope analyses, and it was reported that the trophic plasticity the isotope range of the hadal amphipods was relatively large for δ^13^C [19]. Although plant debris might contribute such extraordinary trophic plasticity, cellulase activity has not been detected in other hadal amphipods to date. Further investigations of the hadal zone will be necessary for understanding the ecology of these deep environments.

## Materials and Methods

### Capturing *H*. *gigas*

On September 10, 2009 we attached four baited traps containing a slice of mackerel to an 11,000 m-class camera system (ASHURA) and lowered it into the deepest point of the Mariana trench (11°22.11′N, 142°25.86′E, depth: 10,897 m) for 2.5 h and captured 185 amphipods, all of which were *H*. *gigas*. The *H*. *gigas* were stored in a −80°C deep freezer or soaked in a methanol:chloroform mixture (1∶1) and stored at 4°C.

### Total organic carbon (TOC) in the sediment

The total carbon (TC) was extracted from 2 g of dried sediment with 100 mM sodium phosphate/5 mM EDTA buffer (pH 8.0), and the TOC was calculated by finding the differences between the TC and the total inorganic carbon. The TC and TOC were measured by combustion oxidation infrared spectrometry according to ISO8245 [45].

### Glucose and disaccharide content of *H*. *gigas*

Five specimens were freeze-dried, crushed and extracted three separate times with 1 ml of distilled water. After removing the insoluble particles by centrifugation (15,000 rpm at 4°C for 10 min), the extract was centrifuged using a 10-KDa cut-off Microcon centrifugal filter device (Millipore Co., Billerica, MA) to remove enzymes and other high molecular weight components. The glucose content was then measured using the Glucose CII Kit (Wako Pure Chemical Industries, Ltd., Osaka, Japan). The maltose content was calculated from the increase in glucose levels after incubation with 1 U of α-glucosidase (Oriental Yeast Co. Ltd., Tokyo, Japan) for 16 h at 37°C. The cellobiose content was calculated from the increase in the glucose content after incubation with β-glucosidase (Oriental Yeast Co. Ltd.) for 16 h at 37°C.

### Measurement of hydrolytic enzyme activities in *H*. *gigas* extracts

The enzymes from crushed *H*. *gigas* were extracted three times with 0.5 ml of distilled water (4°C). All of the enzymatic activities were measured at 30°C. The protease activity was measured by a modified Anson assay in which one unit of activity was defined as the amount of extract required to hydrolyze Hammersten casein to produce a color equivalent to that of 1 µmole of tyrosine in a minute at pH 5.6 [46]. The amylase activity was detected using iodine after incubating the extract with 1% (w/v) soluble starch at a pH of 5.6. One unit was defined as the amount of extract that hydrolyzed soluble starch to cause a 1% decrease in the absorbance at 620 nm in a minute. The cellulase activity was measured with a cellulase assay kit with 1% (w/v) Azo-CM-Cellulose (Megazyme, Wicklow, Ireland). One unit was defined according to the manufacturer's protocol. The cellulase activity of the extracts during HGcel purification was measured as the glucose production from CMC. One unit of cellulase activity was defined as the amount that was needed to hydrolyze enough CMC to produce 1 µg of glucose in a minute. The mannanase activity was measured as the amount of reducing sugar detected by the dinitrosalicylic acid (DNS) assay after a reaction with 0.2% (w/v) glucomannan at pH 5.6 [47]. One unit was defined as the amount of extract required to hydrolyze glucomannan to produce 1 µmol of reducing sugar in a minute. The xylanase activity was measured with an endo-β-xylanase assay kit (Megazyme). One unit was calculated from the activity of the control xylanase (*Trichoderma longibrachiatum*) included in the kit. The alpha-glycosidase activity was measured as the amount of glucose produced with 1% (w/v) maltose at a pH of 5.6. One unit of this enzyme hydrolyzes maltose to produce 1 µmol of glucose in a minute at a pH of 5.6. All of the enzymatic activities were calculated based on the protein content of the sample solutions. The protein content was measured by the Bradford assay using bovine serum albumin as the standard [48].

### TLC analysis of oligosaccharides

Soluble starch (1.0% w/v) was incubated with *H. gigas* extracts at 40°C in a 50 mM sodium acetate buffer (pH 5.6). Samples were taken at intervals and boiled for 5 min. The products were analyzed using TLC with a butanol/acetic acid/water (2∶1∶1 v/v/v) solvent.

### Microscopic observation of gut contents

We examined the gut contents of the *H. gigas* using a stereomicroscope (X20, Nikon Co., Tokyo, Japan). The contents were aspirated using a syringe, suspended in 10 µl of DDW, and then observed using a microscope (X300, Olympus Co., Tokyo, Japan).

### Cellulase reaction using sawdust as substrate

Live oak sawdust purchased from the Adachi Sawmill was washed twice with water, autoclaved at 121°C for 15 min, washed twice with deionized distilled water (DDW) and then dried in air at room temperature. The dried sawdust was suspended in a sodium acetate buffer (pH 5.6) at a concentration of 5% (w/v) and incubated with purified enzyme preparations (0.38 U) at 35°C. The digestion of the sawdust was measured by determining the glucose concentration in the aqueous phase of the reaction mixture.

### Purification of HGcel

We describe the purification of HGcel from a pool of 10 *H*. *gigas*. The amphipods were crushed on ice and centrifuged (1,000 xg for 10 min at 4°C), and the supernatant was collected. Next, 10 ml of ice-cold DDW was added, the sample was vortexed, and the supernatant was collected after centrifugation (1,000 xg for 10 min at 4°C). This step was repeated twice. Saturated ammonium sulfate was added to the pooled supernatants to a final concentration of 30% (w/v). After the extract was incubated on ice for 30 min, it was centrifuged (8,000 xg for 30 min at 4°C), the supernatant was collected and adjusted to a concentration of 60% ammonium sulfate, and the sample was incubated on ice for 3 min. The precipitate demonstrating cellulase activity was collected by centrifugation (8,000 xg for 30 min at 4°C) and suspended in 2 ml of DDW. A 2-ml aliquot was desalted and concentrated to 50 µl using Amicon Ultra 50K columns (Millipore Co.). Next, 50 µl of the enzyme suspension was applied to a 1-ml HiTrap Q Sepharose anion exchange column (Amersham Pharmacia Biotech, Piscataway, NJ), equilibrated with 20 mM sodium phosphate buffer (pH 6.8) and sequentially eluted with 3 ml of the 20 mM sodium phosphate buffer (pH 6.8) buffer containing 0.1 M increments of NaCl to a final salt concentration of 0.6 M. Cellulase activity was detected in the 0.5 M-NaCl fraction, which was diluted with DDW, concentrated to 50 µl using Amicon Ultra 50K columns, applied to the abovementioned equilibrated column and eluted as described above but to a final salt concentration of 0.5 M. The cellulase-containing fractions were collected, washed with DDW, concentrated to 50 µl using Amicon Ultra 50K columns, and then transferred onto a DEAE-TOYOPEARL anion exchange column (TOSOH Co., Tokyo, Japan) (10 mM Tris-HCl (pH 8.6)). The column was eluted sequentially with buffer containing 0.2 M steps of 0–0.6 M NaCl. The cellulase activity was measured based on the amount of glucose produced after a reaction with 1% (w/v) CMC (pH 5.6). The purity of the final enzyme preparation was assessed by SDS polyacrylamide gel electrophoresis (Fig. 3A).

### DNA extraction, PCR amplification and sequencing

15 individual amphipods were immersed in chloroform upon collection. DNA was extracted from 1 g of these amphipods using the DNeasy Blood & Tissue Kit (QIAGEN, Hilden, Germany) according to the manufacturer's instructions. Bacterial 16S rDNA was used as the template for PCR using the universal primer pair ‘Bac27f (5′-AGAGTTTGATCCTGGCTCAG-3′) and Bac1492r (5′-GGTTACCTTGTTACGACTT-3′)’, and archaeal 16S rDNA was used as the template for PCR using the universal primer pair ‘Arch21F (5′-TTCCGGTTGATCCYGCCGGA-3′) and Arch958R (5′-YCCGGCGTTGAMTCCAATT-3′)’. PCR amplification in a 25 µl reaction volume was performed using the GeneAmp PCR System 9700 (Applied Biosystems, Carlsbad, CA) with Speedstar-HS DNA polymerase (Takara Bio Inc., Otsu, Japan) and the buffer supplied with the enzyme. The PCR conditions were as follows: an initial incubation at 96°C for 30 s, 30 cycles consisting of 98°C for 5 s, an incubation at 55°C for 10 s and then another incubation 72°C for 15 s, followed by a final extension step at 72°C for 2 min. The PCR products were analyzed by electrophoresis on a 1% agarose gel, purified using Exo-SAP digestion with Exonuclease I (USB Corp., Cleveland, OH) and shrimp alkaline phosphatase (SAP) (Promega, Fitchburg, WI) at 37°C for 20 min and then treated at 80°C for 30 min to inactivate the enzymes. The PCR products were sequenced using the primers described above and the DYEnamic ET Dye Terminator reagent (GE Healthcare Life Sciences, Piscataway, NJ) on a MegaBACE 1000 (Amersham Biosciences, Piscataway, NJ) automatic sequencer. The nucleotide sequences were trimmed, assembled, and translated using Sequencher 3.7 software (Gene Codes Corp., Ann Arbor, MI).

### Internal amino acid sequence of purified HGcel

HGcel was partially purified from 10 individual amphipods (60% ammonium sulfate precipitation followed by DEAE-Toyo pearl anion exchange chromatography as described above). The cellulase-containing fractions were collected, pooled, desalted, and concentrated. The cellulase preparation was subjected to SDS-PAGE and visualized by staining with Coomassie Brilliant Blue R-250 (Sigma-Aldrich Co., St. Louis, MO). The single 59-kDa band observed was excised from the gel and digested with trypsin. The tryptic peptides were analyzed using the LC-MS/MS system (HPLC: Paradigm MS2, Michrom BioResources, Inc., Aubum, CA; MS: Q-Tof 2 Waters Micromass, Waters Corp., Milford, MA). All of the MS data were analyzed using the Mascot Server (Matrix Science Ltd., London, UK).

## Supporting Information

Figure S1 **An 11,000 m class free-fall camera-soil sampling system ‘ASHURA’ (panel A) and bate traps attached to side bars (panel B).** The ASHURA was equipped a camera and three cores samplers to collect soil sample. Three bait traps were attached to side bars. The bait traps contained filleted mackerel. We lowered ASHURA to the bottom of the Challenger Deep, and traced its position using sonar.
(TIF)

Figure S2 **Microscopic observation of gut contents in** ***H. gigas*** **.** Main contents were some pieces of mackerel tissue (panel A). Arrow indicates stick-like material found in the gut of *H. gigas* (panel B). Bars: 100 µm.
(TIF)

Figure S3 **Plant debris in a core sampler.** Arrow indicates a peace of plant debris. There was more plant debris in another core sampler. Bar showed 5 mm.
(TIF)

Figure S4 **Final step of HGcel purification with DEAE-TOYOPEARL.** HGcel was eluted from 0. 5 ml of DEAE-TOYO PEARL equilibrated with 10 mM Tris-HCl buffer (pH 8.6). HGcel was eluted sequentially with 0.2 M steps of 0–0.6 M NaCl. The volume of each elution buffer was 1.5 ml, and fraction was about 0.1 ml. Cellulase activity was measured with glucose concentration as described in Methods. No. 40 fraction contained cellulase activity, and showed only one protein band in SDS-PAGE (Figure 3A).
(TIF)

Figure S5 **Production of glucose from a piece of plain paper by HGcel.** HGcel was spotted on a piece of plain paper (arrow indicated, left). Glucose was detected as pink colored spot by Glucose CII kit after 15 h incubation at room temperature (arrow indicated, right).
(TIF)

Figure S6 **PCR assay for bacterial or archeal 16S ribosomal DNA in** ***H. gigas*** **.** Mitochondrial cytochrome oxidase subunit I (CYO) DNA was used as a positive control for PCR reaction, and *Escherichia coli* HB101 and *Halobacterium salinarium* ATCC29341 DNAs were used as positive controls for bacteria and archea. M: DNA marker.
(TIF)

Movie S1 **The capture of** ***H. gigas*** **by net style bait trap at the deepest point at 10,898** **m on 20 May 1998.** We baited the trap on the previous day. *H. gigas* can be seen gathering around the bait trap. The remotely operated submersible ‘Kaiko’ collected the trap.
(MOV)
