# Supplementary figures and images for: The Hadal Amphipod Hirondellea gigas Possessing a Unique Cellulase for Digesting Wooden Debris Buried in the Deepest Seafloor
Source: PLoS One. 2012 Aug 15;7(8):e42727. doi: 10.1371/journal.pone.0042727 (PMC3419748; doi:10.1371/journal.pone.0042727)

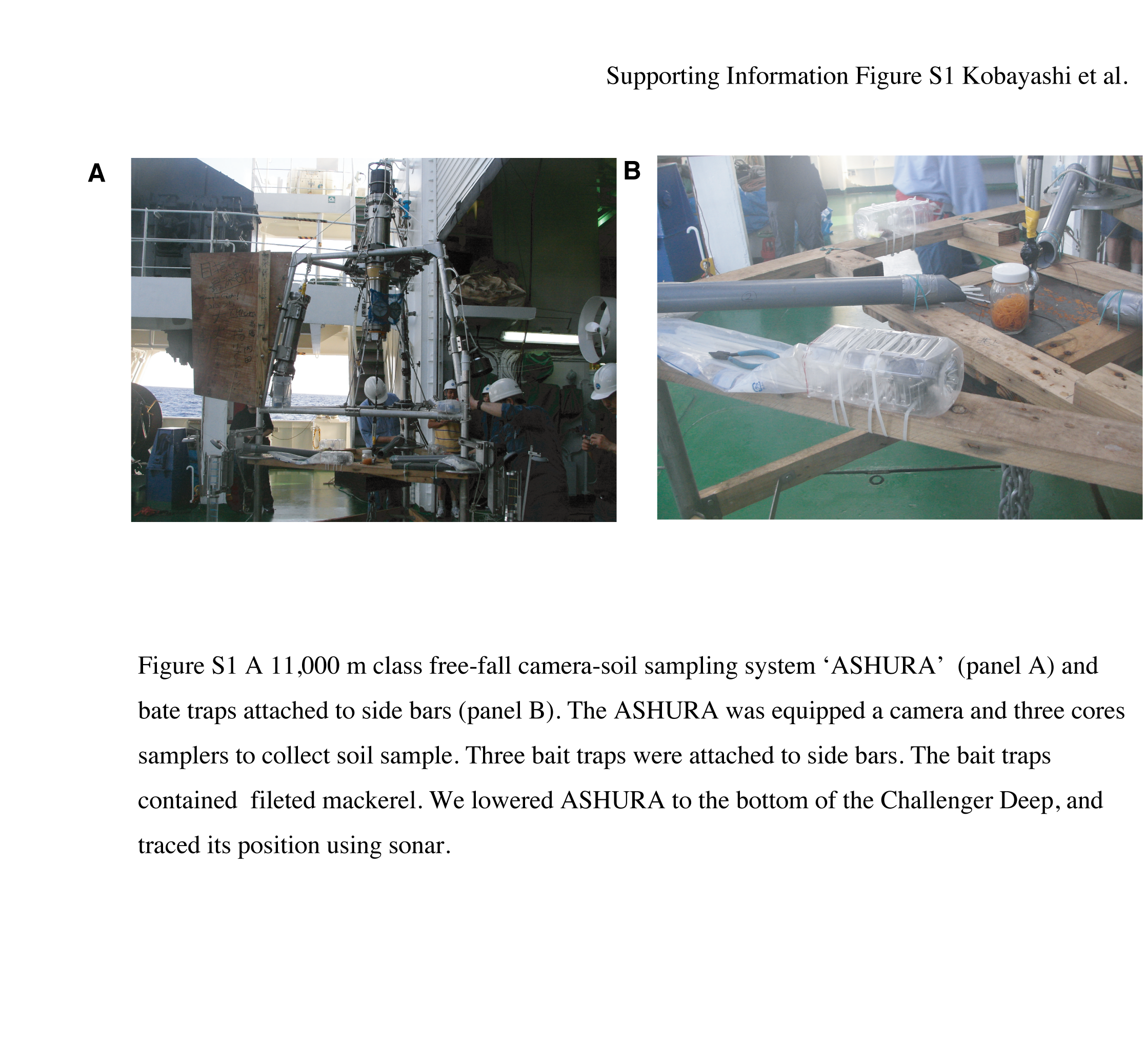

Supplement: Figure S1 — An 11,000 m class free-fall camera-soil sampling system ‘ASHURA’ (panel A) and bate traps attached to side bars (panel B). The ASHURA was equipped a camera and three cores samplers to collect soil sample. Three bait traps were attached to side bars. The bait traps contained filleted mackerel. We lowered ASHURA to the bottom of the Challenger Deep, and traced its position using sonar. (TIF) [file pone.0042727.s001.tif]

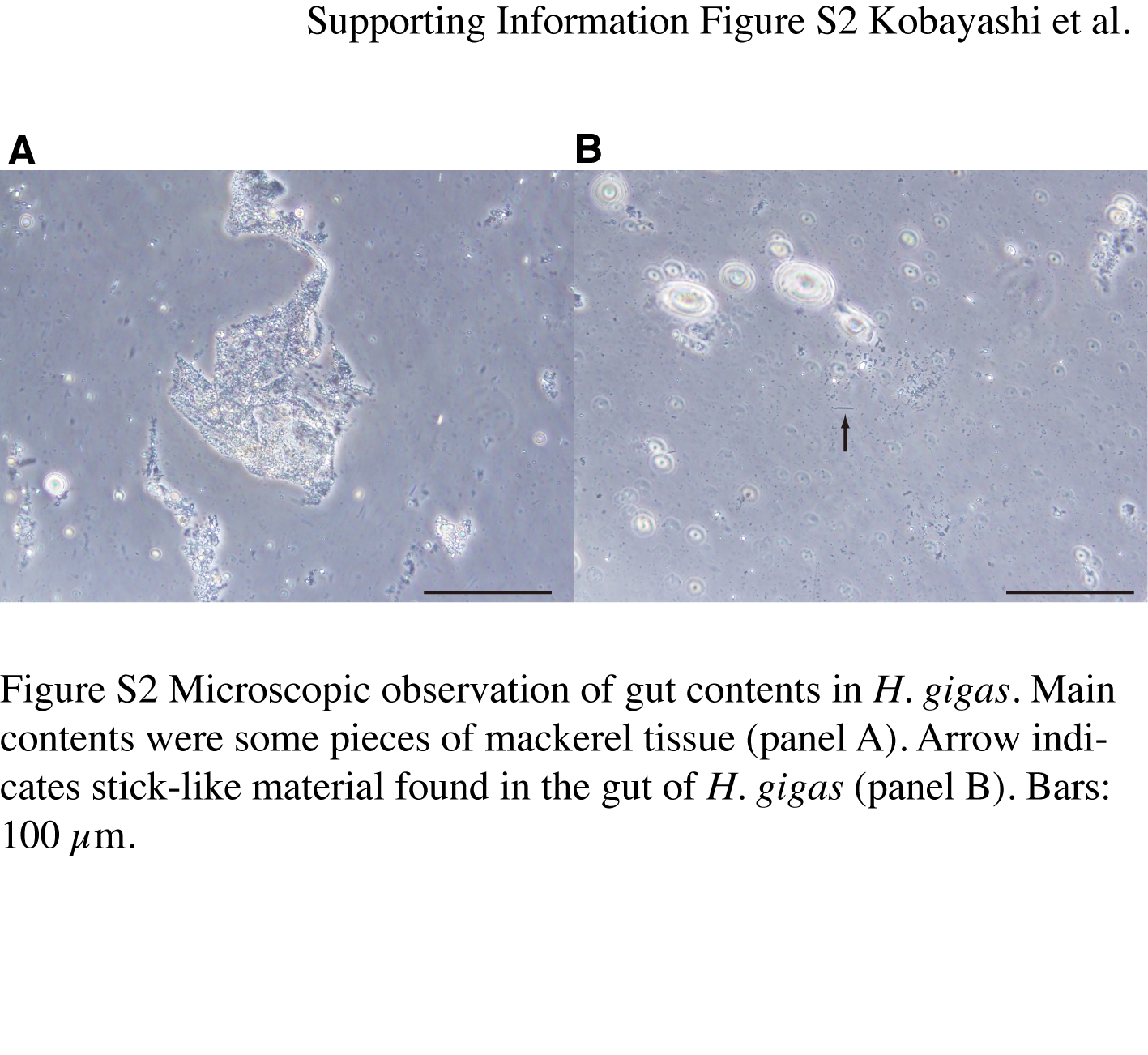

Supplement: Figure S2 — Microscopic observation of gut contents in H. gigas . Main contents were some pieces of mackerel tissue (panel A). Arrow indicates stick-like material found in the gut of H. gigas (panel B). Bars: 100 µm. (TIF) [file pone.0042727.s002.tif]

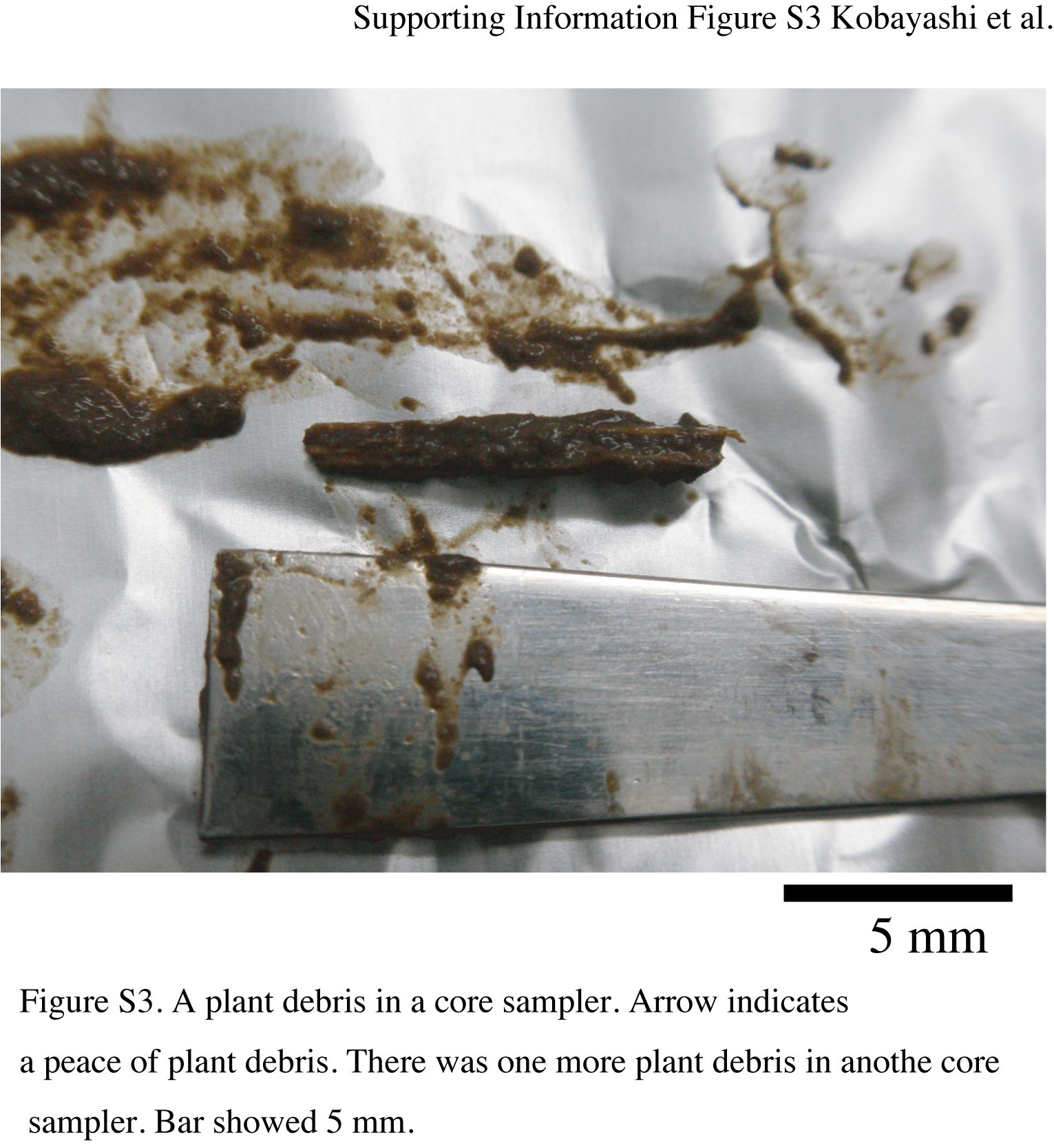

Supplement: Figure S3 — Plant debris in a core sampler. Arrow indicates a peace of plant debris. There was more plant debris in another core sampler. Bar showed 5 mm. (TIF) [file pone.0042727.s003.tif]

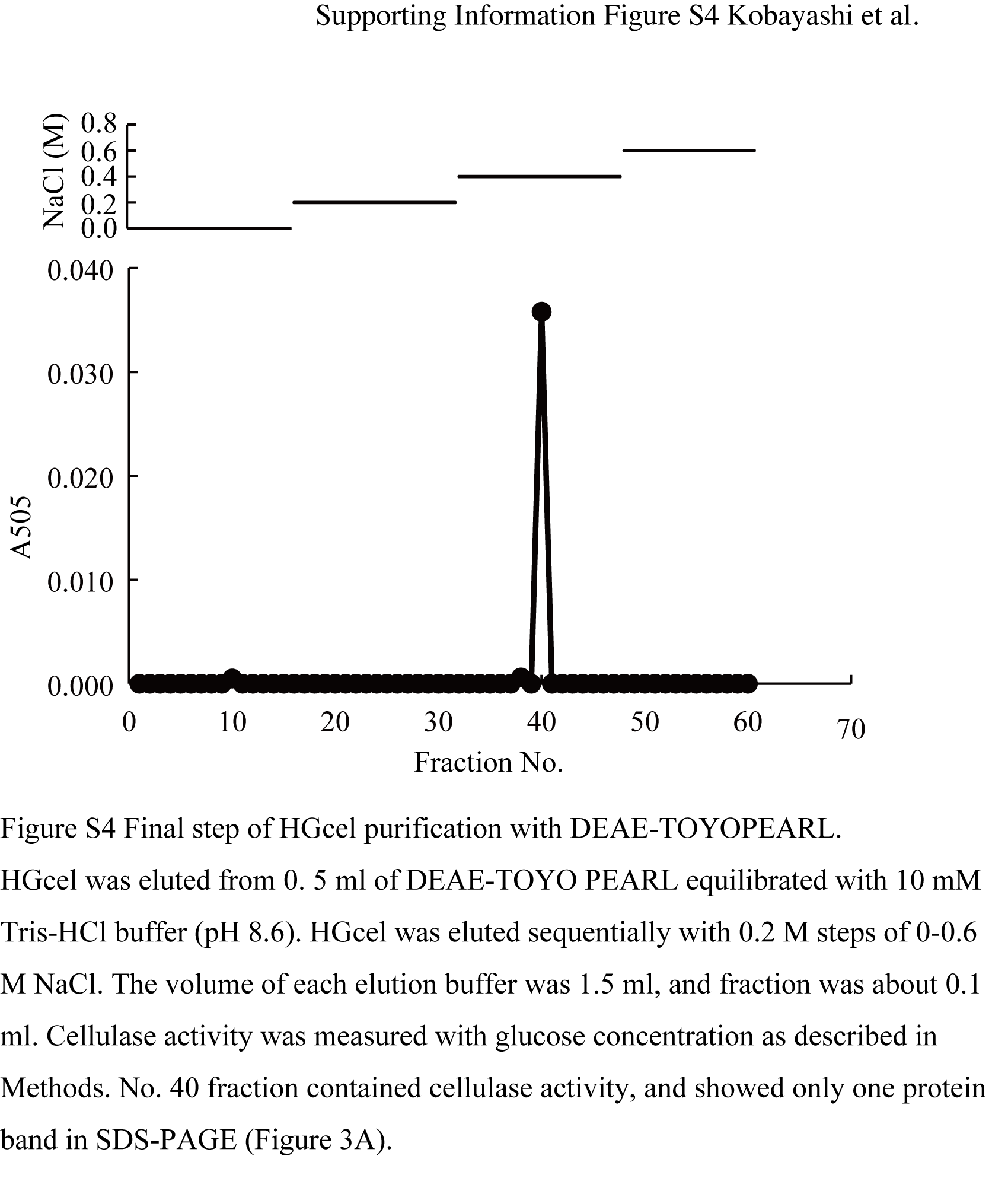

Supplement: Figure S4 — Final step of HGcel purification with DEAE-TOYOPEARL. HGcel was eluted from 0. 5 ml of DEAE-TOYO PEARL equilibrated with 10 mM Tris-HCl buffer (pH 8.6). HGcel was eluted sequentially with 0.2 M steps of 0–0.6 M NaCl. The volume of each elution buffer was 1.5 ml, and fraction was about 0.1 ml. Cellulase activity was measured with glucose concentration as described in Methods. No. 40 fraction contained cellulase activity, and showed only one protein band in SDS-PAGE (Figure 3A). (TIF) [file pone.0042727.s004.tif]

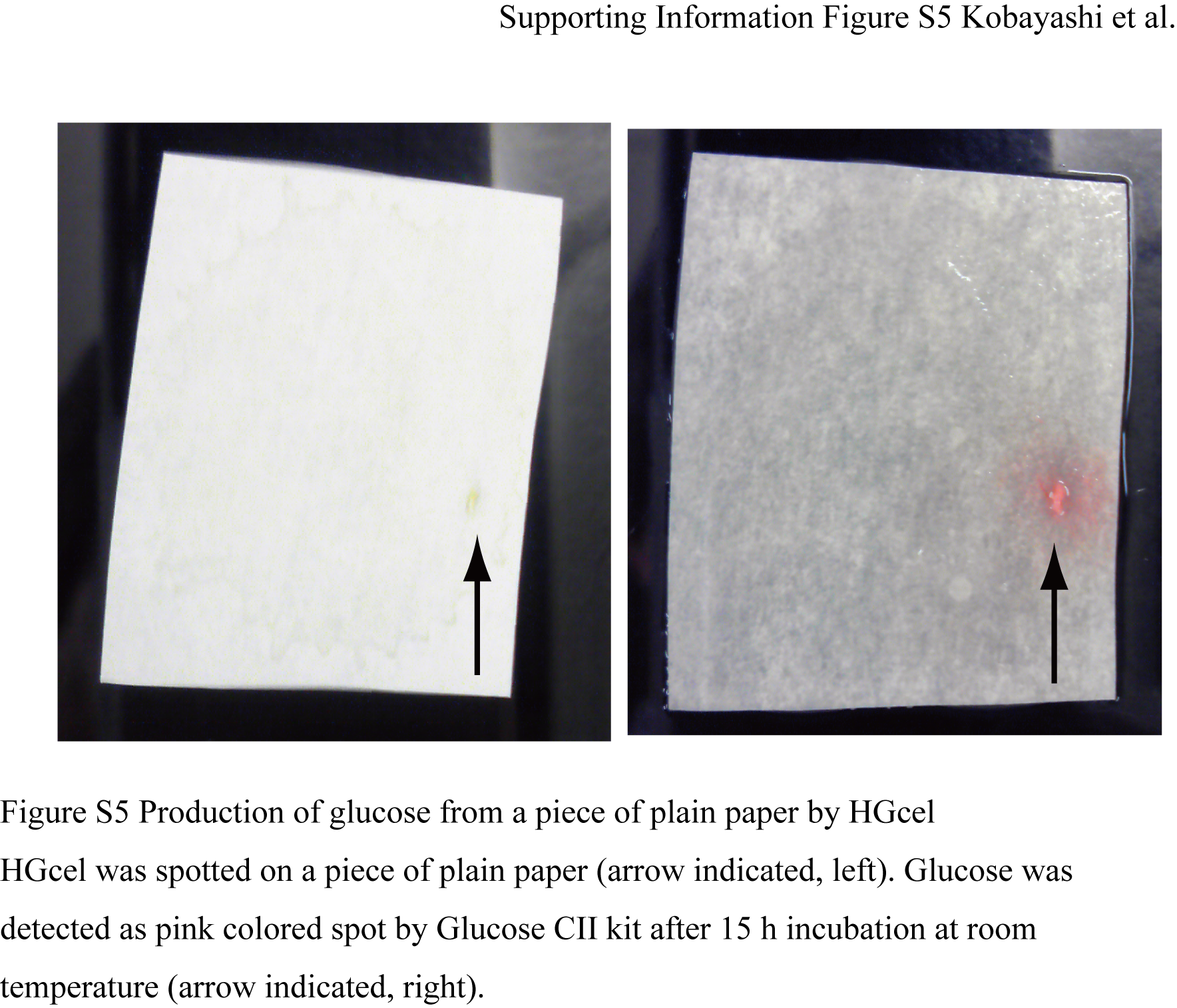

Supplement: Figure S5 — Production of glucose from a piece of plain paper by HGcel. HGcel was spotted on a piece of plain paper (arrow indicated, left). Glucose was detected as pink colored spot by Glucose CII kit after 15 h incubation at room temperature (arrow indicated, right). (TIF) [file pone.0042727.s005.tif]

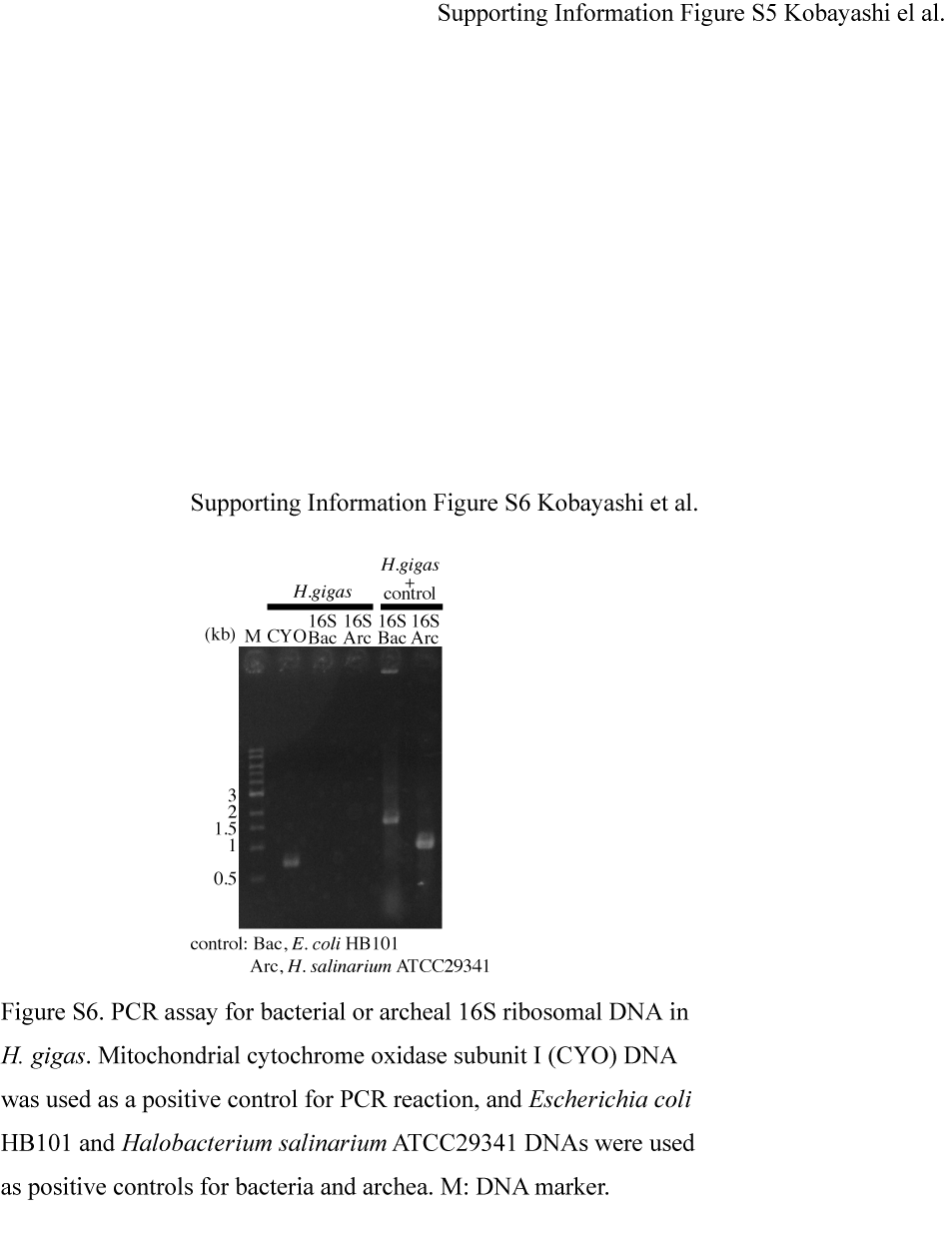

Supplement: Figure S6 — PCR assay for bacterial or archeal 16S ribosomal DNA in H. gigas . Mitochondrial cytochrome oxidase subunit I (CYO) DNA was used as a positive control for PCR reaction, and Escherichia coli HB101 and Halobacterium salinarium ATCC29341 DNAs were used as positive controls for bacteria and archea. M: DNA marker. (TIF) [file pone.0042727.s006.tif]
